# Is transition between subtypes of pemphigus possible? A series of pemphigus vulgaris patients showing the transition to pemphigus foliaceus^☆^

**DOI:** 10.1016/j.abd.2022.09.012

**Published:** 2023-06-23

**Authors:** Rifkiye Kucukoglu, Tugba Atci, Gizem Pinar Sun

**Affiliations:** Department of Dermatology and Venereology, Istanbul University Faculty of Medicine, Istanbul, Turkey

**Keywords:** Autoimmune diseases, Pemphigus, Skin diseases, vesiculobullous

## Abstract

**Background:**

Pemphigus vulgaris (PV) and pemphigus foliaceus (PF) are subtypes of pemphigus with distinct clinical and laboratory features. The transition between these two subtypes has rarely been reported previously.

**Methods:**

The data of PV patients who exhibited clinical and immunoserological transition to PF during the follow-up period were retrospectively evaluated regarding their demographical, clinical, and laboratory characteristics.

**Results:**

Among 453 patients diagnosed with PV, 13 (2.9%) patients exhibited clinical and immunoserological transition from PV to PF. The mean age of PV patients at the time of diagnosis was 39.8 ± 14.7 (19‒62) years and 7 (53.8%) of them were female. These patients showed clinical and immunoserological transition from PV to PF after a period ranging from 4 months to 13 years (mean 36.2 ± 41 months). In addition to typical clinical features of PF, all patients had positive anti-desmoglein-1 and negative anti-desmoglein-3 antibody levels after the clinical transition had occurred without any mucosal involvement. During a mean 7.8 ± 5.8 (2‒21) years of follow-up period after the transition from PV to PF, only one female patient had experienced a re-transition to PV characterized by a relapse of disease involving mucosal surfaces with positive anti-desmoglein-3 antibody levels following a 5-year period of remission period without treatment.

**Study limitations:**

Single-center study with a retrospective study design.

**Conclusion:**

Our series is the largest group of patients reported to show the transition from PV to PF to date with a long follow-up period. The reason behind the disappearance of anti-desmoglein-3 antibodies and the pathogenesis of this phenomenon is not yet elucidated.

## Introduction

Pemphigus Vulgaris (PV) and Pemphigus Foliaceus (PF) are two major subtypes of pemphigus with different clinical and histopathological features as well as anti-Desmoglein (Dsg) antibody profiles. PV differs from PF by mucosal involvement and suprabasal acantholysis and is subdivided into a mucosal dominant type (mPV) that has anti-Dsg3 antibodies and a mucocutaneous subtype (mcPV) that displays both anti-Dsg1 and anti-Dsg3 antibodies. PF patients present with exclusive skin involvement with subcorneal acantholysis caused by anti-Dsg1 antibodies.^1^, ^2^

The transition between these two subtypes of pemphigus has rarely been reported in the last 30 years. The transition from PV to PF is rare in which the underlying immunological changes are not well understood. To the best of our knowledge, only 36 cases of switching from PV to PF were described in the literature until today.^2^, ^3^, ^4^, ^5^, ^6^, ^7^, ^8^, ^9^, ^10^, ^11^, ^12^, ^13^, ^14^, ^15^, ^16^, ^17^ In this study, we aimed to describe the clinical and immunoserological features of 13 patients showing the transition from PV to PF during their disease course with a long follow-up period of up to 21 years following the transition and a detailed literature review about this topic.

## Materials and methods

Medical files of patients diagnosed with PV in autoimmune bullous diseases outpatient clinic in the tertiary center between 1987 and 2021 were retrospectively analyzed. The diagnosis of pemphigus patients was based on clinical, histopathological, Direct Immunofluorescence Microscopy (DIF), Indirect Immunofluorescence (IIF) and Dsg Enzyme-Linked Immunosorbent Assay (ELISA) where available. The data of patients regarding the demographical information, PV subtype (mPV and mcPV), applied treatments before transition, time to transition, and follow-up data after the transition was recorded. The transition from PV to PF was defined based on both the clinical findings and anti-Dsg ELISA levels when available.

The study was approved by the institutional ethical committee and conducted in accordance with the Declaration of Helsinki (Approval number: E-29624016-050.99-358339).

## Results

From a total of 453 patients diagnosed with PV in the department, 13 (2.9%) patients exhibited clinical and immunoserological transition from PV to PF during their follow-up period. Demographical data, clinical, histopathological, and serological features of these patients are shown in Table 1.

**Table 1:** Demographic, clinical, histopathological, serological features of patients showing transition from pemphigus vulgaris to pemphigus foliaceus

Patient	Age (at diagnosis)/Sex	Initial diagnosis	Histopathology	Treatment (before transition)	Initial serological profile	Final diagnosis	Serological profile after transition	Time to transition (month)	Time after transition (years)
1	46/M	Mucocutaneous PV	Suprabasal cleavage	CS, MMF, plasmapheresis	IIF+	PF	Dsg1+	24	13
							Dsg3-		
2	19/M	Mucocutaneous PV	Suprabasal cleavage	CS, AZA	IIF+	PF	Dsg1+	24	21
							Dsg3-		
3	62/M	Mucocutaneous PV	Suprabasal cleavage	CS, AZA, MMF	Dsg1+	PF	Dsg1+	4	2
					Dsg3+		Dsg3-		
4	51/M	Mucocutaneous PV	Suprabasal and intraepidermal cleavage	CS, AZA	Dsg1+	PF	Dsg1+	5	13
					Dsg3+		Dsg3-		
5	21/F	Mucocutaneous PV	Suprabasal cleavage	CS	Dsg1+	PF	Dsg1+	156	3
					Dsg3+		Dsg3-		
6	22/F	Mucocutaneous PV	Suprabasal cleavage	CS, AZA	Dsg1+	PF	Dsg1+	36	7
					Dsg3+		Dsg3-		
7	39/M	Mucocutaneous PV	Suprabasal and intraepidermal cleavage	CS, AZA	Dsg1+	PF	Dsg1+	24	4
					Dsg3+		Dsg3-		
8^a^	32/F	Mucocutaneous PV	Suprabasal and intraepidermal cleavage	CS	Dsg1+	PF	Dsg1+	60	2
							Dsg3-		
9^b^	42/F	Mucocutaneous PV	Suprabasal and intraepidermal cleavage	CS, AZA	Dsg1+	PF	Dsg1+	22	11
					Dsg3+		Dsg3-		
10	50/F	Mucocutaneous PV	Suprabasal cleavage	CS, AZA	Dsg1+	PF	Dsg1+	15	11
					Dsg3+		Dsg3-		
11	22/F	Mucocutaneous PV	Suprabasal cleavage	CS, AZA, Dapsone	Dsg1+	PF	Dsg1+	84	10
					Dsg3+		Dsg3-		
12	62/F	Mucocutaneous PV	Suprabasal and intraepidermal cleavage	CS, AZA	Dsg1+	PF	Dsg1+	12	3
					Dsg3+		Dsg3-		
13	49/M	Cutaneous PV	Suprabasal cleavage	CS	Dsg1+	PF	Dsg1+	5	2.5
					Dsg3+		Dsg3-		

AZA, Azathioprine; CS, Corticosteroid; Dsg1, anti-Desmoglein-1 IgG; Dsg3, anti-Desmoglein-3 IgG; F, Female; IIF, Indirect Immunofluorescence; M, Male; MMF, Mycophenolate Mofetil; PV, Pemphigus Vulgaris; PF, Pemphigus Foliaceus.

a Patient 8 had positive Dsg1 levels but Dsg3 could not be tested upon diagnosis; Dsg testing was not possible at the time of presentation of patients 1 and 2.

b Patient 9 showed re-transition from PF to PV.

Six male and seven female PV patients with a mean age of 39.8 ± 14.7 (19‒62) years at the time of diagnosis were included in this study. Twelve patients were diagnosed with mcPV and only one patient with cutaneous PV with characteristic clinical, histopathological, and immunoserological features (Table 1). Ten patients had positive anti-Dsg-1 and anti-Dsg3 levels detected with ELISA at the time of PV diagnosis, one patient had positive Dsg1 levels but anti-Dsg3 could not be tested. However, this patient had mcPV clinically and showed suprabasal and intraepidermal cleavage histopathologically. Unfortunately, the Dsg ELISA test was not available at the time of presentation of the two other patients who had the longest follow-up period in the present study (13 and 21 years) (Table 1). During follow-up, all these patients were observed to clinically showed the transition from PV to PF after a duration ranging from four months to 13 years (mean: 36.2 ± 41 months). In addition to typical clinical features of PF, all patients had positive anti-Dsg1 and negative anti-Dsg3 levels after the clinical transition had occurred without any mucosal involvement. During a mean 7.8 ± 5.8 (2‒21) years follow-up period after the transition from PV to PF, only one female patient had experienced a re-transition to PV characterized by a relapse of disease involving mucosal surfaces with positive anti-Dsg3 antibody levels following a 5-year period of remission period without treatment.

## Discussion

The two major subtypes of pemphigus, PV and PF, have their own distinct clinical, histological, and immunological characteristics.^1^, ^6^ Transition from PV to PF is an uncommon phenomenon and to the best of our knowledge, only 36 cases are reported in the literature (Table 2).^2^, ^3^, ^4^, ^5^, ^6^, ^7^, ^8^, ^9^, ^10^, ^11^, ^12^, ^13^, ^14^, ^15^, ^16^, ^17^ In this retrospective study, we evaluated a large series of PV patients of whom 13 (2.9%) showed a transition from PV to PF including their data of a long follow-up period after transition up to 21 years.

**Table 2:** Literature review of patients showing transition from pemphigus vulgaris to pemphigus foliaceus

Study/Year	Patient number/Ratio	Age range/Sex	Initial antibody profile	Treatments until transition	Time until transition (years)	Investigations confirming transition/antibody profile after transition	Follow-up after transition (years)/re-transition (n=)
Iwatsuki et al., 1991^3^	2/31	36‒58/F	NA	CS, AZA	1‒3	Hp, IB/Dsg1+^a^ (n = 1)	5‒6/-
Hashimoto et al., 1991^4^	3/NA	NA	NA	NA	3‒20	Hp, IB/Dsg1+^a^	NA
Kawana et al.,1994^5^	2/NA	46‒57/F	Dsg3+^a^	CS	3	Hp, IB/Dsg1+^a^	NA
Chang et al., 1997^6^	1/NA	47/M	NA	CS	3	Hp, IB/Dsg1+^a^, Dsg3+^a^	NA
Mendiratta et al., 2000^7^	1/NA	46/F	NA	CS, CYC	NA	Hp	NA
Komai et al., 2001^8^	4/NA	38‒65/NA	Dsg1+^b^, Dsg3+^b^ (n = 3), Dsg3+^a^ (n = 3)	NA	0.5‒1	Hp, ELISA, IB/Dsg1+^b^ (n = 4), Dsg1+^a^ (n = 3)	NA/Yes (n = 1)
Kimoto et al., 2001^9^	1/NA	77/F	Dsg3+^b^	CS	5	Hp, ELISA/Dsg1+^b^	NA
Tsuji et al., 2002^10^	1/NA	55/M	Dsg3+^b^	CS, PHP	3	Hp, ELISA/Dsg1+^b^	NA
Harman et al., 2002^11^	1/NA	44/F	Dsg3+^b^, Dsg1+^b^	CS, CYC AZA, MMF	5	ELISA/Dsg1+^b^	4/-
Tóth et al., 2002^12^	1/NA	28/M	Dsg3+^b^, Dsg1+^b^	CS	2	Hp, ELISA/Dsg1+^b^	NA
Ng et al., 2005^13^	3/NA	29, 56/M	NA	CS, AZA	2‒4	ELISA/Dsg1+^b^	1‒7/-
		45/F					
Levy-Sitbon et al., 2012^14^	1/NA	47/M	Dsg3+^b^, Dsg1+^b^	CS, rituximab	2.7	Hp, ELISA/Dsg1+^b^	NA
Espana et al., 2014^15^	2/NA	35, 49/M	Dsg3+^a^, Dsg1+^a^	CS, AZA, CYC, rituximab	2‒3.8	Hp, ELISA, IB-IP/Dsg1+^a^ (n = 2), Dsg3+^a^ (n = 1)	NA
Ito et al., 2016^16^	1/NA	57/M	Dsg3+^b^, Dsg1+^b^	CS, IVIG	0.2	Hp, ELISA/Dsg1+^b^	NA
Chapman et al., 2019^17^	1/NA	56/M	NA	AZA, rituximab	1.2	Hp/NA	NA
Mohammadrezaee et al., 2020^2^	12/1483	24‒79/6M, 6F	Dsg3+^b^, Dsg1+^b^	NA	0.5‒5.3	ELISA/Dsg1+^b^	NA/Yes (n = 1)
Present study, 2022	13/453	19‒62/6M,7F	Dsg3+^b^, Dsg1+^b^ (n = 10), NA (n = 3)	CS, MMF, AZA, PHP, dapsone	0.3‒13	ELISA/Dsg1+^b^	2‒21/Yes (n = 1)

AZA, Azathioprine; CS, Corticosteroid; Dsg1, anti-Desmoglein-1 IgG; Dsg3, anti-Desmoglein-3 IgG; F, Female; Hp, Histopathology; IB, Immunoblot; IP, Immunoprecipitation; M, Male; MMF, Mycophenolate Mofetil; NA, Non-available; PV, Pemphigus Vulgaris; PF, Pemphigus Foliaceus; CYC, Cyclophosphamide; PHP, Plasmapheresis.

a Immunoblot.

b ELISA, Enzyme Linked Immunosorbent Assay.

Conventionally, the clinical phenotype of PV and PF remains unchanged in the course of clinical progression in each patient, and the transition from PV to PF is an uncommon event (Table 2).^2^, ^3^, ^4^, ^5^, ^6^, ^7^, ^8^, ^9^, ^10^, ^11^, ^12^, ^13^, ^14^, ^15^, ^16^, ^17^ Time until transition ranges from 1.5 months to 20 years among the previously reported cases whereas, in the present study, it ranged from four months to 13 years. In addition, there was not any sex predilection regarding the transition from PV to PF both in the present study (M/F: 0.9) and literature (M/F: 1.1) (Table 2).^2^, ^3^, ^4^, ^5^, ^6^, ^7^, ^8^, ^9^, ^10^, ^11^, ^12^, ^13^, ^14^, ^15^, ^16^, ^17^

Although the possibility of transition from PV to PF or vice versa, has been mentioned since the 1960s,^18^, ^19^ in 1991 Iwatsuki et al. were the first to report two women among 31 patients with pemphigus, showing a transition from PV to PF with the changes in clinical features and histological findings which is supported by immunoblot analysis in one of these cases.^3^ Kawana et al. reported the first investigation completely demonstrating changes in antigen profiles at the molecular level with immunoblotting analysis consistent with a transition from PV to PF.^5^ However, since immunoblotting is not a quantitative technique, it may not be a good option to show this transition, as it can lead to incorrect results.^12^ To our knowledge, the first study showing the correlation between the clinical transition from PV to PF and the changes in the autoantibody profile demonstrated by the ELISA system, in addition to immunofluorescence and immunoblotting, was reported in 2001.^8^ Interestingly, in that study, three patients were described as having concomitant features of PV and PF which is a less frequently described phenomenon, five patients showed a transition from PV to PF (two of them were previously reported elsewhere),^5^ one patient showed the transition from PV (anti-Dsg1-3 positive in ELISA, anti-Dsg3 positive in immunoblot) to PF (anti-Dsg1 positive both in ELISA and immunoblot) and subsequently showed re-transition to PV (anti-Dsg1 positive and anti-Dsg3 positive in ELISA, anti-Dsg3 positive in immunoblot) in one year period. ^8^ In the present study, only one female patient showed the transition from PV to PF after 22 months of diagnosis and then showed re-transition from PF to PV following a 5-year period of remission period without treatment. Additionally, in some studies, in the follow-up period of patients showing a transition from PV to PF, re-transition to PV has been observed.^2^, ^8^ Interestingly, among previously reported patients showing the transition from PV to PF, two of them had elevated levels of both anti-Dsg1 and anti-Dsg3 antibodies after the transition.^6^, ^15^ Although subtype conversion among pemphigus patients is extremely rare, the transition from PF to PV is even rarer (Table 2). The reason behind this trend is yet to be elucidated and needs further evaluation in future studies.

Epitope spread phenomenon and HLA restriction were suggested as possible explanations behind the inter-subtype conversion of pemphigus patients.^8^ Epitope spread phenomenon can be described as a process resulting in tissue damage which leads to the exposal of immunologically hidden certain protein components to the immune system evoking a secondary autoimmune response.^9^, ^20^ In PV, both intramolecular epitope spreading, leading to recognition of multiple epitopes within Dsg3, as well as intermolecular epitope spreading, from Dsg3 to Dsg1, have been shown to occur. In addition, both intramolecular and intermolecular epitope spreading may be associated with the progression of the PV from pure mPV to mcPV.^17^, ^20^ The biochemical similarity between Dsg1 and Dsg3 has raised the hypothesis that an epitope spread phenomenon may play a role in the transition between PV and PF.^6^, ^20^ However, the epitope spreading hypothesis cannot be applied to patients showing the transition from PF to PV and in these patients, the pathomechanism remains still controversial.^16^, ^20^ In addition, the reason behind the disappearance of anti-Dsg3 antibodies in patients showing the transition from PV to PF, as seen in the present cases, is still unknown.^10^ In one study, it was shown that extracellular domains of Dsg3 are altered, leading to a reactivity loss in the patients switching from PV to PF with immunoblotting-immunoprecipitation analysis.^15^ The mechanism underlying this alteration is yet to not be elucidated. In addition, previously, it was argued whether the transition from PV to PF is permanent or transient. It is a matter of debate whether treatment cessation leads to the reproduction of anti-Dsg3 antibodies which were previously masked in the patients showing the transition to PF under treatment, resulting in a relapse where PV phenotype with mucosal involvement recurs, as seen in one of our patients.^11^, ^13^ Chang et al. reported a patient showing autoantibodies against both Dsg3 and Dsg1 after transitioning from PV to PF, which was supported clinically and histologically. The authors claimed that pathogenic autoantibodies shifted from Dsg3 antibodies to Dsg1 antibodies.^6^ This transition means the antigenicity loss of anti-Dsg3. The epitope spreading hypothesis cannot be applied to these cases.^16^

## Conclusion

The transition between the subtypes of pemphigus is an unexpected phenomenon and reports of such cases are extremely rare. The present case series of 13 patients is the largest group of patients reported to show a transition from PV to PF to date with a long follow-up period. The transition was confirmed both with characteristic clinical features and serologic profiles. Little about the mechanism of the clinical and immunoserological transition from PV to PF is known. Better clarification of the pathogenesis and additional immunological studies are required to elucidate the transition between pemphigus subtypes in further studies.

## Financial support

None declared.

## Authors' contributions

Rifkiye Kucukoglu: Approval of the final version of the manuscript; critical literature review; data collection, analysis, and interpretation; effective participation in research orientation; intellectual participation in propaedeutic and/or therapeutic management of studied cases; manuscript critical review; preparation and writing of the manuscript; statistical analysis; study conception and planning.

Tugba Atci: Approval of the final version of the manuscript; critical literature review; data collection, analysis, and interpretation; effective participation in research orientation; intellectual participation in propaedeutic and/or therapeutic management of studied cases; manuscript critical review; preparation and writing of the manuscript; statistical analysis; study conception and planning.

Gizem Pinar Sun: Approval of the final version of the manuscript; critical literature review; data collection, analysis, and interpretation; effective participation in research orientation; intellectual participation in propaedeutic and/or therapeutic management of studied cases; manuscript critical review; preparation and writing of the manuscript; statistical analysis; study conception and planning.

## Conflicts of interest

None declared.

## References

[1] Ujiie I., Ujiie H., Iwata H., Shimizu H. (2019). Clinical and immunological features of pemphigus relapse. Br J Dermatol..

[2] Mohammadrezaee M., Etesami I., Mahmoudi H., Lajevardi V., Tavakolpour S., Balighi K. (2020). Transition between pemphigus vulgaris and pemphigus foliaceus: a 10-year follow-up study. J Dtsch Dermatol Ges..

[3] Iwatsuki K., Takigawa M., Hashimoto T., Nishikawa T., Yamada M. (1991). Can pemphigus vulgaris become pemphigus foliaceus?. J Am Acad Dermatol..

[4] Hashimoto T., Konohana A., Nishikawa T. (1991). Immunoblot assay as an aid to the diagnoses of unclassified cases of pemphigus. Arch Dermatol..

[5] Kawana S., Hashimoto T., Nishikawa T., Nishiyama S. (1994). Changes in clinical features, histologic findings, and antigen profiles with development of pemphigus foliaceus from pemphigus vulgaris. Arch Dermatol..

[6] Chang S.N., Kim S.C., Lee I.J., Seo S.J., Hong C.K., Park W.H. (1997). Transition from pemphigus vulgaris to pemphigus foliaceus. Br J Dermatol..

[7] Mendiratta V., Sarkar R., Sharma R.C., Korann R.V. (2000). Transition of pemphigus vulgaris to pemphigus foliaceus. Indian J Dermatol Venereol Leprol..

[8] Komai A., Amagai M., Ishii K., Nishikawa T., Chorzelski T., Matsuo I. (2001). The clinical transition between pemphigus foliaceus and pemphigus vulgaris correlates well with the changes in autoantibody profile assessed by an enzyme-linked immunosorbent assay. Br J Dermatol..

[9] Kimoto M., Ohyama M., Hata Y., Amagai M., Nishikawa T. (2001). A case of pemphigus foliaceus which occurred after five years of remission from pemphigus vulgaris. Dermatology..

[10] Tsuji Y., Kawashima T., Yokota K., Tateish Y., Tomita Y., Matsumura T. (2002). Clinical and serological transition from pemphigus vulgaris to pemphigus foliaceus demonstrated by desmoglein ELISA system. Arch Dermatol..

[11] Harman K.E., Gratian M.J., Shirlaw P.J., Bhogal B.S., Challacombe S.J., Black M.M. (2002). The transition of pemphigus vulgaris into pemphigus foliaceus: a reflection of changing desmoglein 1 and 3 autoantibody levels in pemphigus vulgaris. Br J Dermatol..

[12] Tóth G.G., Pas H.H., Jonkman M.F. (2002). Transition of pemphigus vulgaris into pemphigus foliaceus confirmed by antidesmoglein ELISA profile. Int J Dermatol..

[13] Ng P.P., Thng S.T. (2005). Three cases of transition from pemphigus vulgaris to pemphigus foliaceus confirmed by desmoglein ELISA. Dermatology..

[14] Lévy-Sitbon C., Reguiaï Z., Durlach A., Goeldel A.L., Grange F., Bernard P. (2013). Transition from pemphigus vulgaris to pemphigus foliaceus: a case report. Ann Dermatol Venereol..

[15] España A., Koga H., Suárez-Fernández R., Ohata C., Ishii N., Irarrazaval I. (2014). Antibodies to the amino-terminal domain of desmoglein 1 are retained during transition from pemphigus vulgaris to pemphigus foliaceus. Eur J Dermatol..

[16] Ito T., Moriuchi R., Kikuchi K., Shimizu S. (2016). Rapid transition from pemphigus vulgaris to pemphigus foliaceus. J Eur Acad Dermatol Venereol..

[17] Chapman C.M., Kwock J., Cresce N., Privette E., Cropley T., Gru A.A. (2019). IgG/IgA pemphigus in a patient with a history of pemphigus vulgaris: An example of epitope spreading?. J Cutan Pathol..

[18] Perry H.O. (1961). Pemphigus foliaceus. Arch Dermatol..

[19] Perry H.O., Brunsting L.A. (1965). Pemphigus foliaceus. Further observations. Arch Dermatol..

[20] Salato V., Hacker-Foegen M.K., Lazarova Z., Fairley J.A., Lin M.S. (2005). Role of intramolecular epitope spreading in pemphigus vulgaris. Clin Immunol..

